# The Civil Hospitals and Dispensaries of the Madras Presidency

**Published:** 1902-12-27

**Authors:** 


### THE CIVIL HOSPITALS AND DISPENSARIES OF THE MADRAS PRESIDENCY.

Few changes took place in the hospitals of the Madras Presidency during 1900. A new hospital was completed at Vániyambádi, containing beds for 12 patients. Up to this time there had been no accommodation in that particular district, except for the relief of out-patients. Four new dispensaries, maintained by local funds, were opened, the one at Kaity in the Nilgiris being of a temporary nature for the relief of the many cases of fever in the valley. It remained in use for five months, and was closed in November when the need ceased to exist. A Church of Scotland Mission dispensary was set up during the year at Sholinghur, in North Arcot, under a lady doctor, and dispensaries were also started in different places by the Baptist Mission, the Canadian Mission, and the Society for the Propagation of the Gospel. The total number of civil hospitals and dispensaries in working order during the year was in all 464, of which 197 were hospitals. The number of patients treated during 1900 was 4,448,881, exceeding by 222,573 the total of the previous year. There was a decrease in the number of Europeans and Eurasians under treatment, but the large increase in other classes included men, women, and children. Strangely enough, notwithstanding this large addition to the patients under treatment, there appears a marked decrease in the number of in-patients—1,794. But this is only an apparent falling off, due to the fact that the figures for police, railway, and private unaided hospitals are now given separately. The results of treatment show that 27,500 were cured, 9,017 relieved, 3,356 discharged otherwise, and 3,442 died. The death-rate rose from 5·42 per cent. in 1899 to 7·70 per cent. in 1900, the result of the great number of cholera cases admitted—almost three times that of the previous year. A total of 137,407 operations were performed during the year, which shows a very marked increase, 16,146 over the number recorded in 1899. Notwithstanding the far larger number of operations performed, the death-rate per cent. was lower. Forty-seven deaths were due to septic infection contracted before, six to septic infection contracted after, admission. The 321 midwives employed in the various institutions of the Presidency attended 25,793 cases of labour. This was 255 more cases than in the previous year, although the number of midwives remained the same. The income of the Civil Medical Institution was 10,20,748 rupees, of which the Government paid 8·69 per cent. The expenditure left a cash balance in hand of 2,948 rupees, the cost of each in-patient being rather more than previously, owing to a longer average detention in hospital.

